# Gr-1 Ab Administered after Bone Marrow Transplantation plus Thymus Transplantation Suppresses Tumor Growth by Depleting Granulocytic Myeloid-Derived Suppressor Cells

**DOI:** 10.1371/journal.pone.0097908

**Published:** 2014-05-21

**Authors:** Ming Shi, Ming Li, Yunze Cui, Yasushi Adachi, Susumu Ikehara

**Affiliations:** 1 Department of Stem Cell Disorders, Kansai Medical University, Hirakata, Osaka, Japan; 2 Department of Occupational and Environmental Health, School of Public Health, Guangdong Medical College, Dongguan, Guangdong, China; 3 JIMRO Co., Ltd., Takasaki, Gunma, Japan; 4 Division of Clinical Pathology, Toyooka Hospital, Toyooka, Hyogo, Japan; Rutgers - New Jersey Medical School, United States of America

## Abstract

It has been shown that allogeneic intra-bone marrow–bone marrow transplantation (IBM-BMT) plus thymus transplantation (TT) is effective in treating recipients with malignant tumors. Although TT increases the percentage of T cells in the early term after BMT, the myeloid-derived suppressor cells (MDSCs) are still the dominant population. We used the Gr-1 Ab to deplete the granulocytic MDSCs (G-MDSCs) in tumor-bearing mice that had received BMT+TT. Two weeks after the BMT, the mice injected with Gr-1 Ab showed smaller tumors than those in the control group. In addition, Gr-1 Ab significantly increased the percentages and numbers of CD4^+^ and CD8^+^ T cells, and decreased the percentages and numbers of MDSCs and G-MDSCs. No side effects of the Gr-1 Ab on recipient or donor thymus were observed. These findings indicate that Gr-1 Ab administered after BMT+TT may enhance the effectiveness of tumor suppression.

## Introduction

Allogeneic bone marrow transplantation (allo-BMT) has been used as a potentially curative therapy for not only leukemias, immunodeficiencies, and autoimmune diseases but also solid malignant tumors. Radiotherapy and/or chemotherapy performed as a conditioning regimen for BMT are prerequisites for suppressing host immunity and to reduce the tumor burden. The conditioning regimen also induces tissue damage and the release of a storm of proinflammatory cytokines. The proinflammatory cytokines include tumor necrosis factor-α (TNF-α), and interleukins 1 and 6, which have been reported to promote the activation and maturation of antigen-presenting cells and the rapid amplification of donor T cells [1], [2]. The therapeutic effects of allo-BMT on malignancies are also mediated via the induction of the graft-versus-tumor effect by immunocompetent cells in the graft. Therefore, we have recently developed an allo-BMT method in conjunction with thymus transplantation (TT). Because we have found that TT using newborn thymus is the most effective method of suppressing tumors, we used newborn thymus in this study. It has also been shown that newborn TT can increase the percentage and number of CD4^+^ T cells in the short term after BMT. The combination of allo-BMT and TT (allo-BMT+TT) is effective in restoring donor-derived T cell function in tumor-bearing mice, and no concomitant graft-versus-host disease (GVHD) was observed.

Myeloid-derived suppressor cells (MDSCs) are a phenotypically heterogeneous cell population that includes myeloid progenitor cells and immature myeloid cells [3]. MDSCs are characterized by their myeloid origin, immature state, and most importantly by their potent ability to suppress different aspects of immune responses, especially T-cell proliferation and cytokine production [4]. Studies have shown that MDSCs accumulate in most patients and experimental animals with cancer [5], [6]. In mice these cells are defined as Gr-1^+^CD11b^+^ cells, and consist of two major subsets: Ly6G^+^Ly6C^low^ granulocytic (G-MDSCs) and Ly6G^−^Ly6C^high^ monocytic (M-MDSCs) cells [7]. Inhibition of tumor growth was observed by depleting the G-MDSCs using the Gr-1 (RB6-8C5) Ab [8]. In this study, we investigate the influence of Gr-1 Ab administration on tumor suppression after allo-BMT+TT.

## Materials and Methods

### Mice

C57BL/6 (B6) and BALB/c mice were purchased from Shimizu Laboratory Supplies (Shizuoka, Japan). 8–12-week-old male mice were used for BMT. For TT, B6 mice were sacrificed one day after birth to obtain newborn thymuses. All the mice were maintained in a specific pathogen-free room. The Committee on the Ethics of Animal Experiments of Kansai Medical University approved our experiments. All protocols (11–142) for these animal experiments were performed in accordance with the Guidelines for Animal Experimentation, Kansai Medical University.

### Inoculation of tumor cells

One day before the inoculation of tumor cells, the recipients (BALB/c mice) underwent total-body irradiation (3 Gy) using a ^137^Cs irradiator (Gammacell 40 Exactor; MDS Nordion International). The next day, Meth-A cells (2×10^5^ in 50 ul PBS) were subcutaneously inoculated into the right flank of these mice.

### Experimental protocol

Ten days after the inoculation of tumor cells, the BALB/c mice were irradiated with 7 Gy. The next day, the bone marrow cells (BMCs) were prepared by flushing them from the medullary cavities of the femurs and tibias of B6 mice with phosphate-buffered saline (PBS). The BMCs (1×10^7^) were then injected directly into the tibial cavity of the recipient mice via the intra-bone-marrow route. For TT and Gr-1 groups, one newborn thymus was simultaneously transplanted under the renal capsule in the recipients with BMT. From Day 5, recipient mice in the Gr-1 and TT groups were injected with 5 ug Gr-1 or its isotype Ab into the peritoneal cavity respectively every other day. The tumor diameter was measured every 2 or 3 days.

### Reagents, flow cytometric analysis and cell number calculation

The antibodies (Abs) used in this study were as follows: purified rat anti-mouse Gr-1 Ab (Biolegend); fluorescein isothiocyanate (FITC) conjugated anti-mouse Gr-1 and H-2K^b^ Ab; phycoerythrin (PE) conjugated anti-mouse H-2K^d^, CD4, CD8 and CD11b Ab; peridinin chlorophyll protein (PerCP)-Cy5.5 conjugated anti-mouse CD45 Ab (BD Pharmingen, San Diego, CA). Samples for flow cytometry were analyzed using a FACSCalibur flow cytometer (BD Biosciences). We counted the total numbers of nuclear cells in the peripheral blood using an SF-3000 autoanalyzer (Sysmex). The numbers of CD4^+^ T cells, CD8^+^ T cells, MDSC and G-MDSCs per ul were calculated by using the total cell number and percentage.

### Statistical analysis

The results are represented as means ± SD. The Student's *t* test was used to determine any statistical significance. A p-value of <0.05 was considered to be a significant difference.

## Results

### Relationship between tumor size and the percentage of MDSCs or G-MDSCs

**Figure 1:**
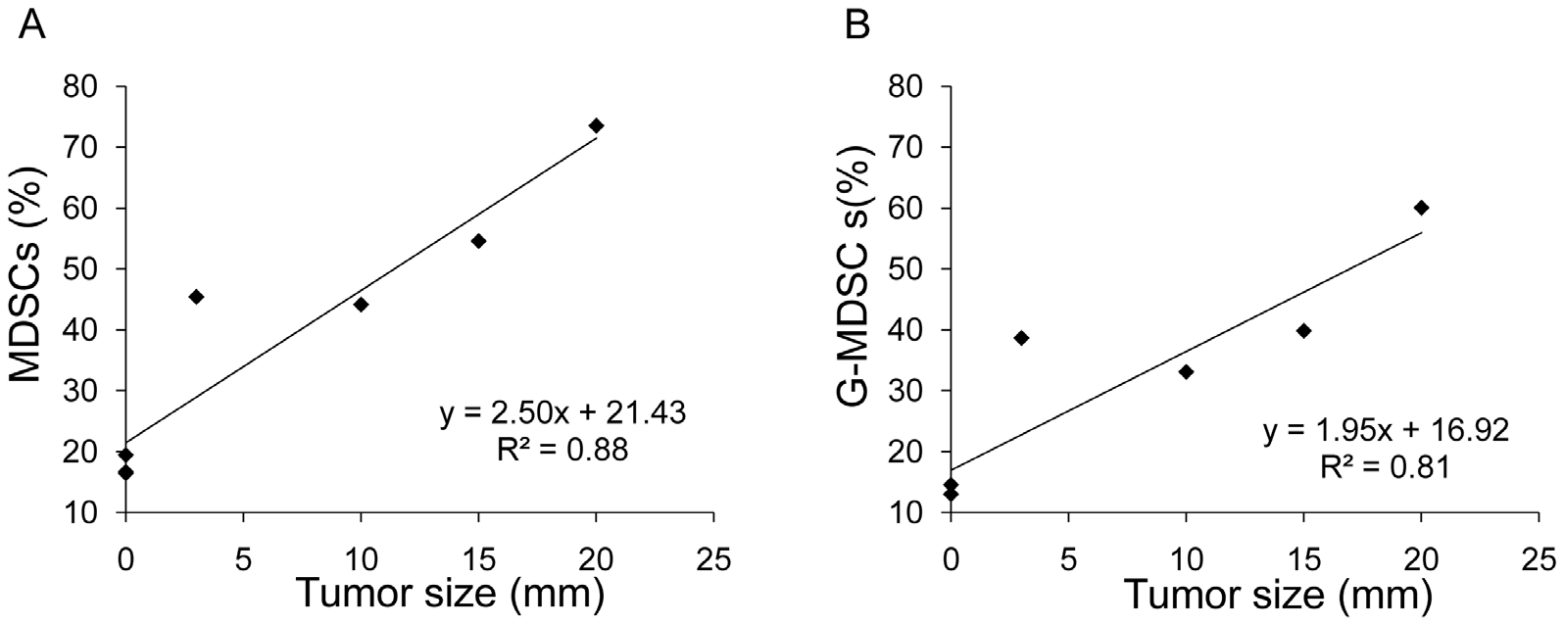
Relationship between tumor size and the percentage of MDSCs or G-MDSCs. BALB/c mice were irradiated with 3 Gy, and Meth-A cells were then subcutaneously inoculated into the right flank of these mice. The percentages of MDSCs (A) and G-MDSCs (B) in the peripheral blood were then analyzed.

To address the relationship between tumor size and MDSCs, Meth-A cells were inoculated into BALB/c mice subcutaneously. MDSCs were defined as Gr-1^+^CD11b^+^ cells. Two weeks later, MDSCs of the peripheral blood were analyzed. The proportion of Gr-1^+^CD11b^+^ cells increased dramatically in mice with larger tumors. A size-dependent percentage of MDSCs was observed in these mice (Fig. 1A). Youn JI et al have reported that the population of G-MDSCs, but not M-MDSCs, was predominant in most tumor models and that it suppressed T cell proliferation [7]. Therefore, we analyzed the percentage of G-MDSCs in the peripheral blood. Tumor bearing mice showed a high proportion of G-MDSCs, and the trend toward increasing numbers of G-MDSCs was consistent with that of MDSCs (Fig. 1B). The evaluated percentages of MDSCs and G-MDSCs in the mice inoculated with Meth-A cells suggested that these cells might be used for suppressing tumor growth.

### G-MDSC-depletion using Gr-1 Ab more effective in suppressing tumors when combined with allo-BMT+TT

**Figure 2:**
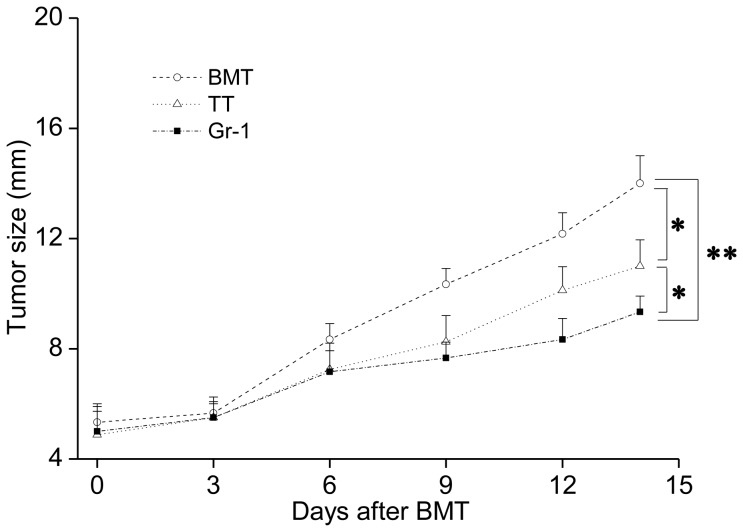
Gr-1 Ab administered after allo-BMT+TT was more effective in suppressing tumor growth. Recipient BALB/c mice with tumors were irradiated with 7 Gy one day before BMT. The next day, these mice were injected with 1×10^7^ B6 BMCs using the IBM-BMT method. For TT and Gr-1 groups, one newborn thymus was simultaneously transplanted under the renal capsule in the recipients that had received BMT. From Day 5, recipient mice in the Gr-1 and TT groups were injected with 5 ug Gr-1 or its isotype Ab respectively every other day. The tumor diameter was measured every 2 or 3 days. *p<0.05, **p<0.01.

We have shown that allogeneic IBM-BMT plus newborn TT is effective in tumor suppression. One of the mechanisms is that TT increases the percentage of CD4^+^ T cells in the short term (5 or 7 days) after BMT. However, no significant effects on CD8^+^ T cells were observed [9], [10]. As reconstitution of the myeloid lineage is much faster than that of the T lineage cells, and G-MDSCs can suppress CD8^+^ T cell proliferation and IFN-γ production, we tried to deplete the G-MDSCs using the Gr-1 Ab [7], [11]. The choice for the optimal dose of Gr-1 Ab was one that would effectively deplete the G-MDSCs from the peripheral blood by about 10 days. Preliminary experiments of different doses of Gr-1 Ab treatment showed that the administration of 5 ug Gr-1 Ab every other day could meet this requirement due to the low cell number in the peripheral blood soon after BMT. From Day 5, some mice that had received the allo-BMT+TT combination were injected with 5 ug Gr-1 (RB6-8C5) Ab every other day. As with our previous results, the tumors were significantly smaller in the mice treated with allo-BMT+TT than in those treated with allo-BMT alone. The mice administered the Gr-1 Ab showed the greatest degree of tumor regression (Fig. 2). Since the use of Gr-1 Ab to deplete G-MDSCs has been reported to inhibit the growth of tumors, these results suggested that Gr-1 Ab could be used not only alone but also combined with allo-BMT+TT [8].

### Gr-1 Ab increased the percentage and number of T cells and decreased the percentage and number of G-MDSCs

**Figure 3:**
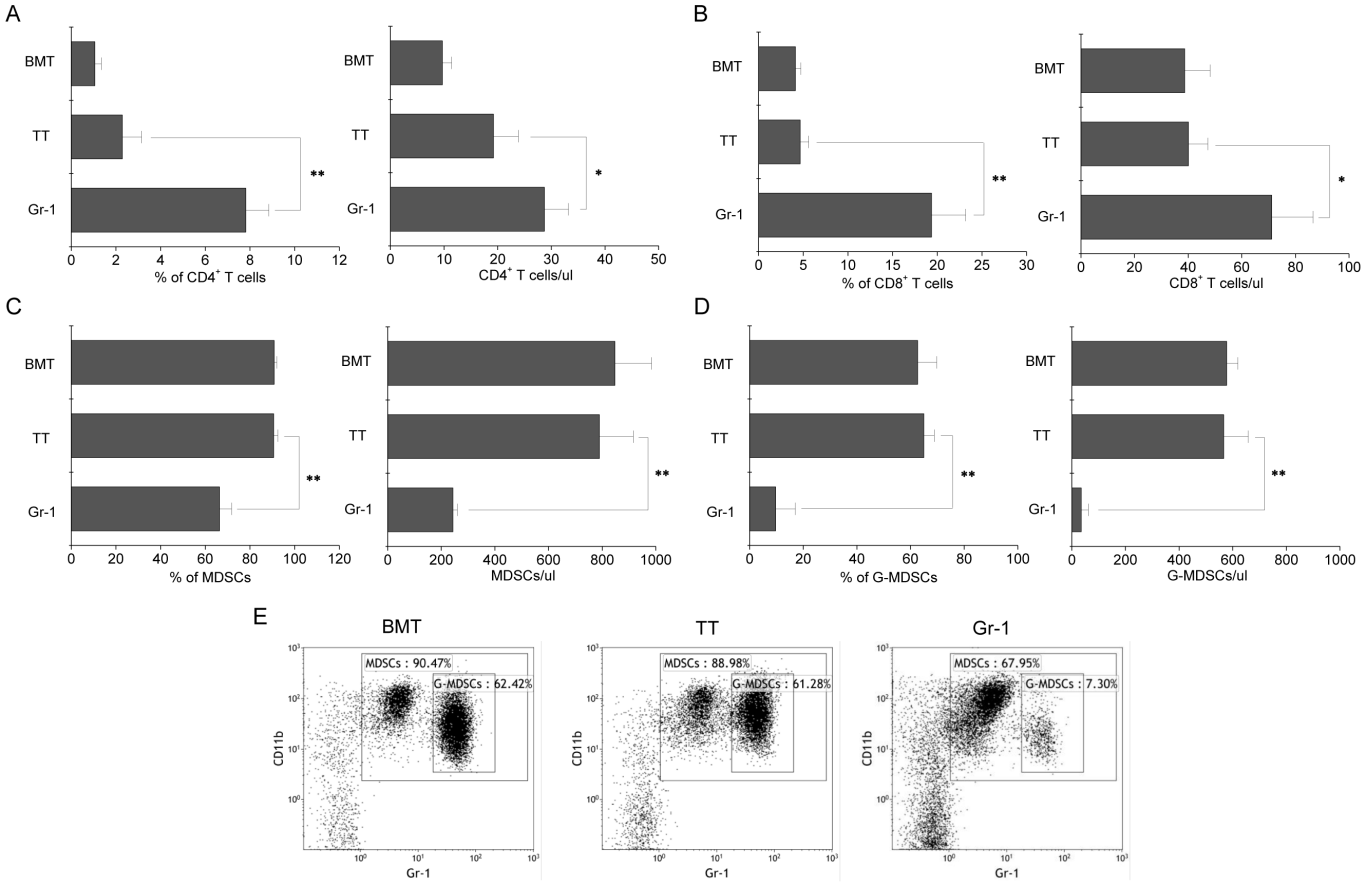
Gr-1 Ab increased the percentages and numbers of CD4^+^ and CD8^+^ T cells, and decreased the percentages and numbers of MDSCs and G-MDSCs two weeks after BMT. Recipient mice were sacrificed two weeks after BMT. The percentages and numbers of CD4^+^ T (A), CD8^+^ T (B), MDSCs (C) and G-MDSCs (D) in the peripheral blood were analyzed by FACS and sysmex. Representative FACS phenotypes of MDSCs (E). The recipients in all groups showed full donor chimerism (>98%, data not shown). *p<0.05, **p<0.01.

The presence of both donor CD4^+^ and CD8^+^ T cells has been reported to be critical in allo-BMT against murine solid tumors [12], [13]. We therefore analyzed the peripheral blood two weeks after BMT and found that the percentages and numbers of CD4^+^ and CD8^+^ T cells in the Gr-1 group were significantly higher than those in the TT and BMT groups (Fig. 3A and B), suggesting that the administration of Gr-1 Ab contributed to the tumor suppression by increasing the percentage and number of T cells. As MDSCs and G-MDSCs have stimulated interest as a therapeutic target as a result of their ability to suppress T cell immune responses, these two subsets were also analyzed [14], [15]. The results showed that the percentages and numbers of MDSCs and G-MDSCs in the Gr-1 group decreased significantly (Fig. 3C–E). These data indicate that the Gr-1 Ab injection ablated, at least transiently, the immune suppressive capacity of the G-MDSCs subsets.

### Gr-1 Ab has no side effects on recipient or transplanted thymus

**Figure 4:**
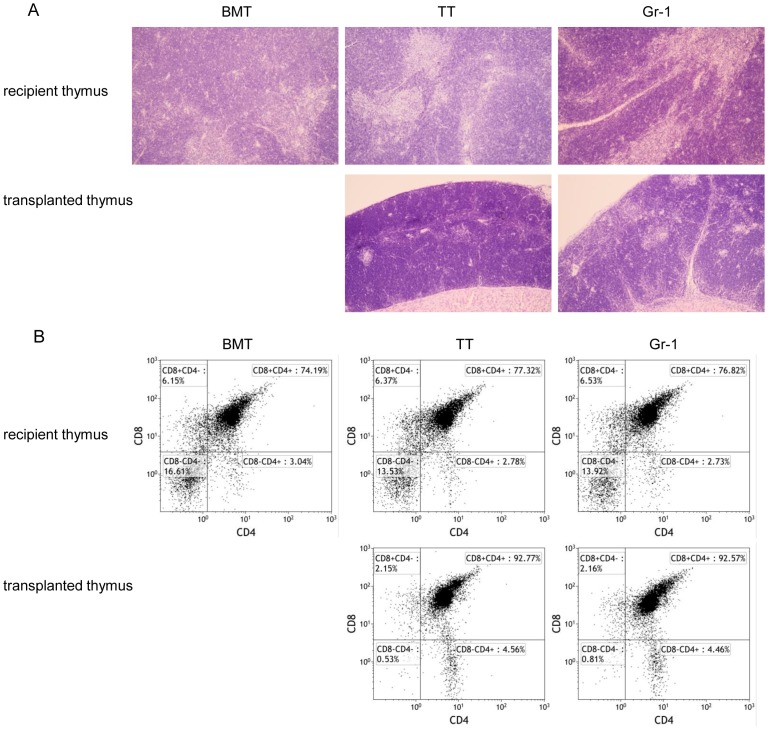
Gr-1 Ab has no side effects on recipient or transplanted thymus. Recipient mice were sacrificed two weeks after BMT. The recipient and donor thymuses were collected for HE staining and FACS analysis. Representative histological findings (A) and FACS phenotypes (B) are shown. Macroscopic findings, original magnification 100×. Cells analyzed for CD4^+^ and CD8^+^ expression were first gated by CD45^+^ cells.

In tumor-bearing mammals, including mice and humans, T-cell function decreases due to involution of the thymus. TT combined with BMT is one strategy to restore T-cell function [16]. Gr-1 Ab improved the percentage of both CD4^+^ and CD8^+^ T cells in the allo-BMT+TT setting. Since factors that affect T cell reconstitution may impair the recovery of recipient thymus and development of transplanted thymus, we examined the effects of Gr-1 Ab on these thymuses [10]. The recipient thymus and transplanted thymus were removed for histological diagnosis and FACS analysis after the peripheral blood had been collected. Recipient and transplanted thymuses in the Gr-1 group were similar to those in the TT group, and histologically displayed well-defined cortical and medullary areas and phenotypically showed similar distribution of CD4^+^ and CD8^+^ T cells two weeks after BMT (Fig. 4A and B). These data suggest that the Gr-1 Ab has no side effects on the recovery of the recipient thymus and the development of the transplanted thymus.

## Discussion

In this study, we examined the anti-tumor effects of Gr-1 Ab administered after allo-BMT+TT. Gr-1 Ab increased the percentages and numbers of CD4^+^ and CD8^+^ T cells, and decreased the percentage and number of G-MDSCs. Tumors in the Gr-1 group were smaller than in the BMT and TT groups.

The conditioning regimen for BMT reduced the tumor burden and produced lots of cytokines that have a wide range of biological effects. Some cytokines, such as TNF-α, have anti-tumor effects and could be of benefit for tumor suppression after BMT [1]. Meanwhile, IL-1 and IL-6 contributed to MDSC accumulation, MDSCs not only suppressing T-cell function but also inducing T-cell apoptosis [17], [18], [19]. The combination of allo-BMT+TT has been proven to be a novel strategy for suppressing tumor growth [16], this strategy restoring T-cell function and improving the percentage of CD4^+^ (but not CD8^+^) T cells in the short term after BMT [9]. Methods to prevent the impairment of MDSCs on T cells after BMT should be explored. Gr-1 Ab is used to reduce the level of G-MDSCs and thus inhibit tumor growth [8]. Therefore we wonder whether the administration of Gr-1 Ab after BMT would improve the anti-tumor effects. Our results showed that the percentage and number of G-MDSCs two weeks after BMT decreased significantly due to the injection of Gr-1 Ab (Fig. 3D). In addition, the percentages and numbers of CD4^+^ and CD8^+^ T cells were increased. These data suggested that Gr-1 Ab benefited TT and tumor suppression by the amelioration of CD8^+^ T cells and the reduction of G-MDSCs. Consequently, more effective suppression of tumor growth was observed in the Gr-1 group (Fig. 3).

Factors that affect the cytokines and T cell reconstitution may influence the recovery of the recipient thymus and the development of the transplanted thymus [10]. However, no side effects of Gr-1 Ab on the thymuses were observed by two weeks after the BMT. The suppressor activity of the G-MDSCs could be transiently ablated by Gr-1 Ab injection, but Gr-1 Ab also triggered myeloid cells in the bone marrow, inducing myelopoiesis [20]. Therefore, the process of Gr-1 Ab administration in our experiment was limited since we only continued observations for two weeks after the BMT. Longer-term observation should thus be explored.

The tumor suppression seen after BMT involves a number of mechanisms. Both T cells and G-MDSCs play important roles in the immune system, and both are reconstituted by donor transplants (from BMCs or TT). The administration of Gr-1 Ab helps to suppresses tumor growth by depleting the G-MDSCs and increasing the percentage and number of T cells, and may thus represent a new choice for more successfully inhibiting tumor growth.
